# Supplementary material for: Role of flying cars in sustainable mobility
Source: Nat Commun. 2019 Apr 9;10:1555. doi: 10.1038/s41467-019-09426-0 (PMC6456499; doi:10.1038/s41467-019-09426-0)
Supplement: Supplementary file 1 — Supplementary Information [file 41467_2019_9426_MOESM1_ESM.pdf]

Role of Flying Cars in Sustainable Mobility

Supplementary Information

Kasliwal et al.

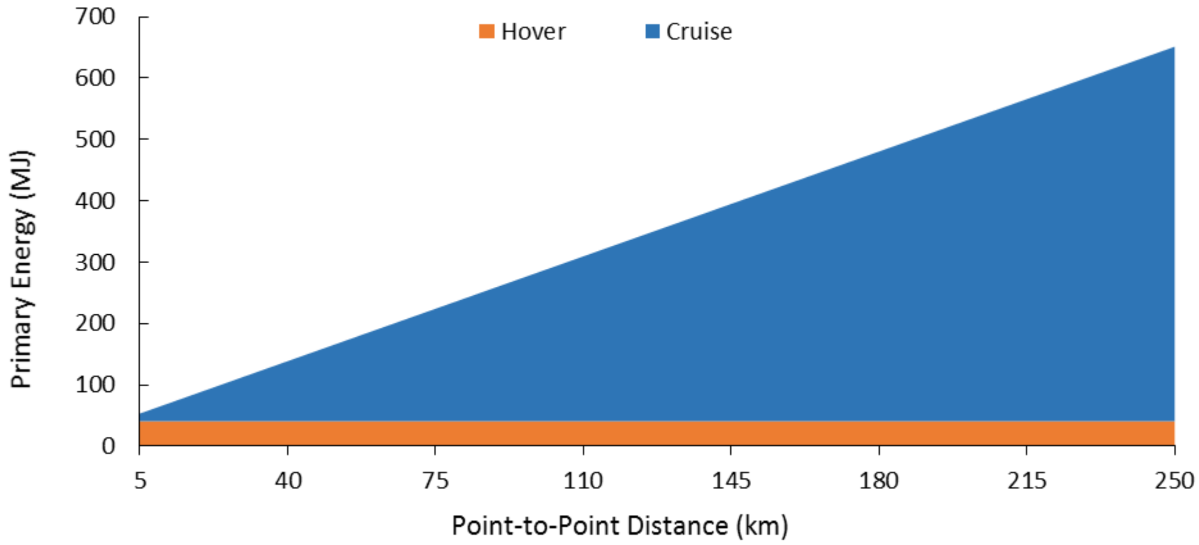

Supplementary Figure 1 | VTOL GHG emissions over a range of trip distances. The GHG emission results for a single-occupant VTOL are broken out by the hover and cruise phases over trip distances from 5 to 250 km. Note that the climb phase is modeled as part of cruise. See the Methods section for details. Also, the takeoff and landing hover phases are combined for simplicity, and the powerless descent phase is omitted since it is assumed to have zero emissions.

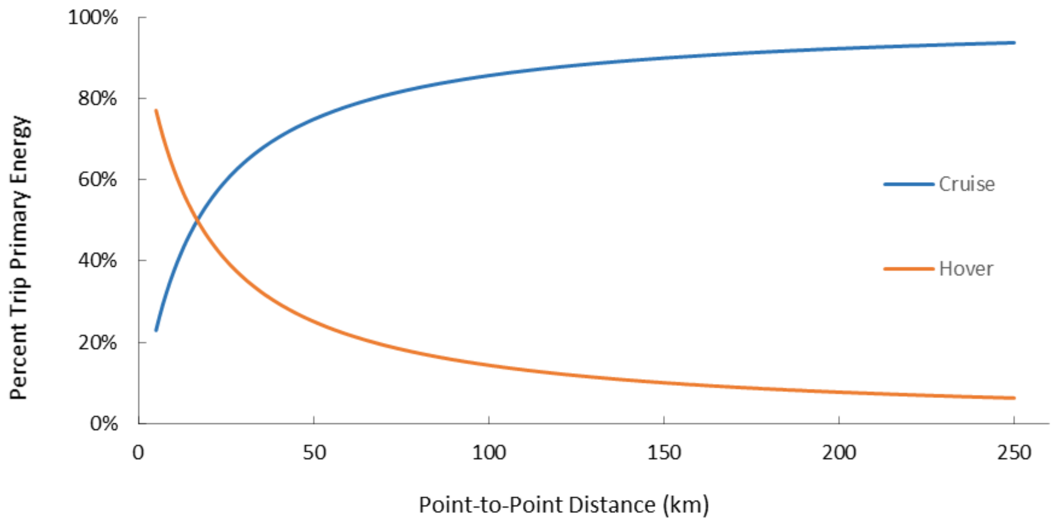

Supplementary Figure 2 | Primary energy use by flight profile phase. The percentage of the total trip primary energy use by flight profile phase is provided over a range of point-to-point trip distance. Hover dominates at smaller distances before cruise takes over around 20 km.

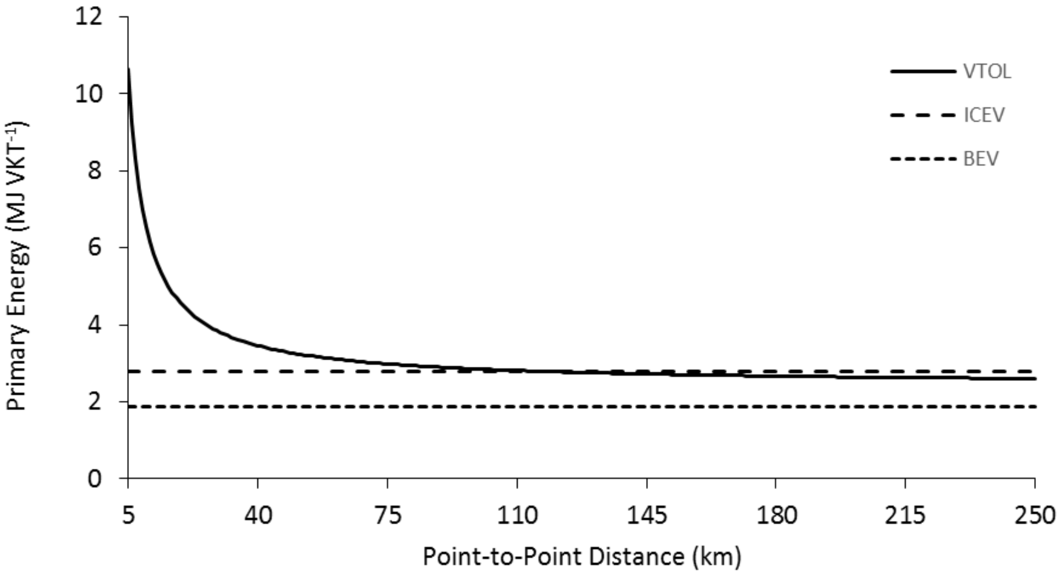

Supplementary Figure 3 | Primary energy normalized by vehicle kilometers traveled. The primary energy results for single-occupant VTOLs and ground-based vehicles (ICEV and BEV) are normalized by vehicle kilometers traveled (VKT). This illustrates the impact of amortizing the fixed burden from the hover phase over longer distances. The VTOL primary energy breaks even with the ICEV at 120 km.

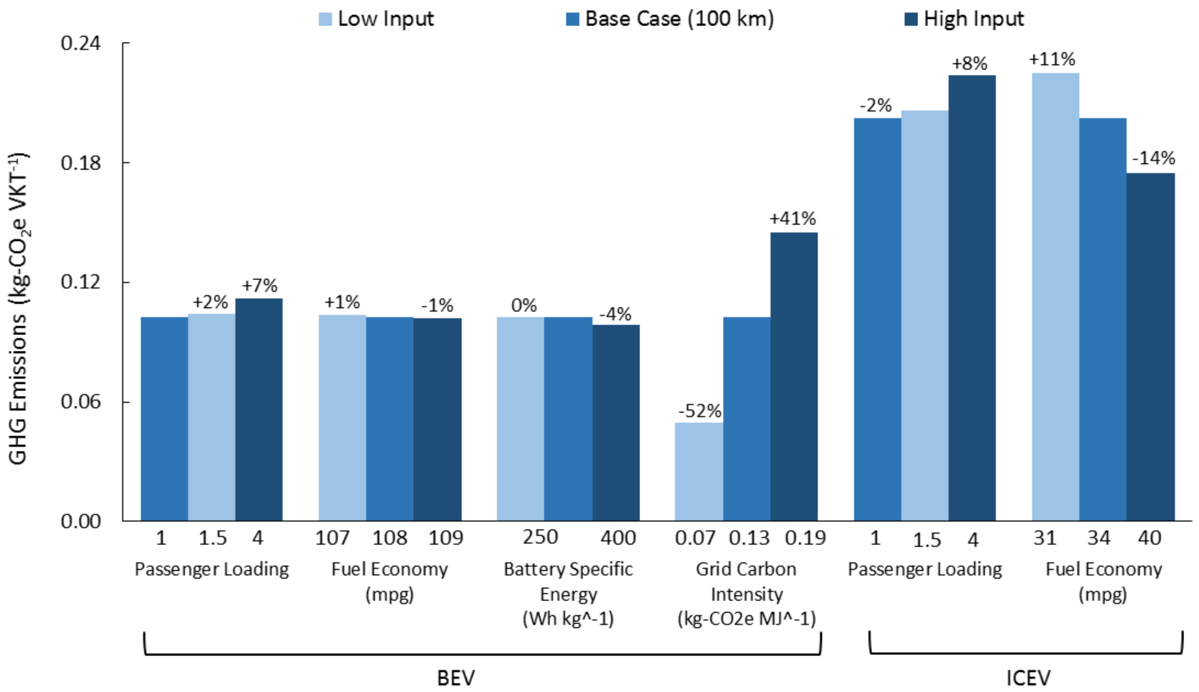

Supplementary Figure 4 | Sensitivity analysis for ground-based vehicles. Six key modeling parameters are individually varied over realistic bounded ranges within the modeling of the 100 km base-case scenarios. Variation in the electrical grid carbon intensity has the largest impact for the BEV, while variation in the fuel economy has the largest impact for the ICEV.

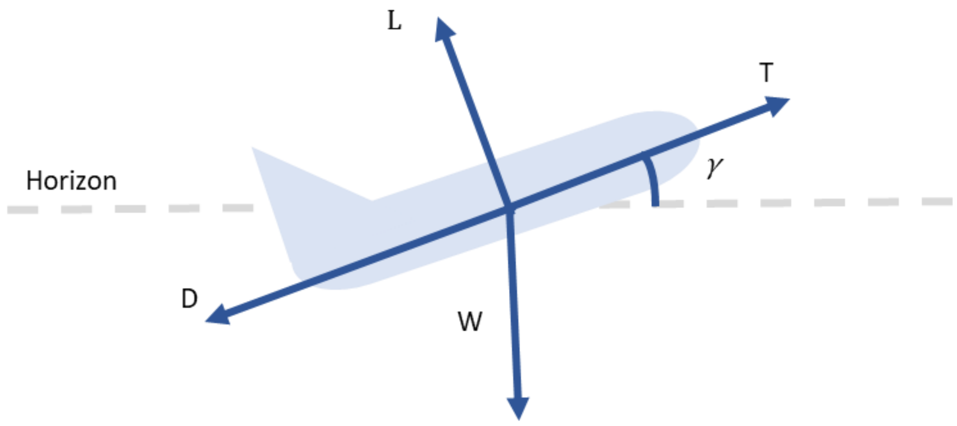

Supplementary Figure 5 | Aircraft free body diagram in steady climb. The free body diagram indicates the relationship between forces during steady climb. These relationships are used in the energy modeling derivations.

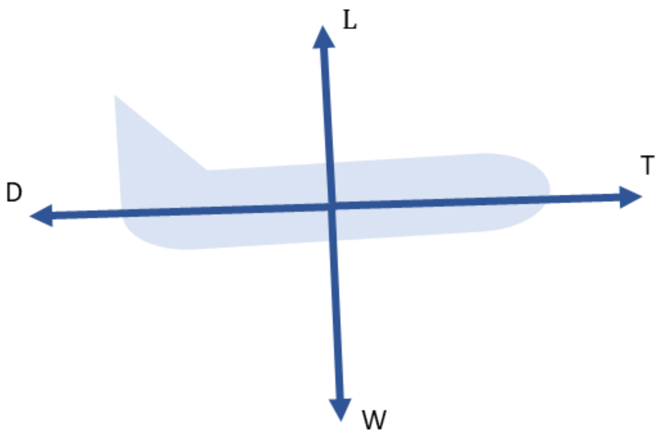

Supplementary Figure 6 | Aircraft free body diagram in steady, level cruise. The free body diagram indicates the relationship between forces during level cruise. These relationships are used in the energy modeling derivations.

Supplementary Table 1: On-road adjusted fuel economies for BEV and ICEV

Variant	On-road adjusted UDDS Fuel Economy	On-road adjusted HWFET Fuel Economy
2020 BEV, LR	304 Wh mi^{-1}	309 Wh mi^{-1}
2020 ICEV	30.7 MPG	39.5 MPG

Supplementary Table 2: Specified test speeds for five-cycle procedure[1]

Bag, Cycle	Average Speed (mph)
Bag 2, FTP	16.1
Bag 3, FTP	25.6
City, US06	21.7
Highway, US06	61.2
HWFET	48.2

Supplementary Note 1

On-road adjusted fuel economy values for both BEVs and ICEVs[2] formed the basis of our fuel economy modeling, and are provided in Supplementary Table 1.

Baseline fuel economy values for the BEV (306.2 Wh mi^{-1}, or 108.4 MPGe) and the ICEV (34.1 MPG) were computed in Supplementary Equation (1) as the harmonically weighted average of a combined drive cycle (55% city/45% highway). [1]

$$Combined\ Fuel\ Economy = \frac{1}{\left(\frac{0.55}{On-road\ adjusted\ UDDS\ Fuel\ Economy}\right)+\left(\frac{0.45}{On-road\ adjusted\ HWFET\ Fuel\ Economy}\right)} \quad (1)$$

Supplementary Note 2

To account for upstream energy impacts of producing grid electricity, a primary energy to delivered electricity factor is applied. For the 2020 U.S. Average Grid, GREET reports a value of 2.0 MJ primary energy per MJ of generated electricity.[3] However, this value does not fully account for the power-plant losses for nuclear-generated electricity, assigning only 0.21 MJ primary energy to 19% of total generation-share contributed by nuclear. To incorporate full impacts, we use the NREL U.S. LCI database.[4] A factor of 3.11 MJ primary energy per MJ of nuclear electricity is applied to the nuclear contribution.

This results in a 2.45 MJ primary energy impact per MJ of electricity delivered, amounting to an efficiency of 40.8%.

Supplementary Note 3

The on-road average velocities for city (20.6 mph) and highway (58.5 mph) driving, adapted from the five-cycle test models for fuel economy[1], are derived using Supplementary Equations (2) and (3), with input parameters specified in Supplementary Table 2. These form the basis for modeling travel time on the ground.

$$On-road\ City\ Driving\ Speed = (0.48 * Bag\ 2\ Speed) + (0.41 * Bag\ 3\ Speed) + (0.11 * US06\ City\ Speed); \quad (2)$$

$$On-road\ Highway\ Driving\ Speed = (0.21 * HWFET\ Speed) + (0.79 * US06\ Highway\ Speed); \quad (3)$$

The combined cycle (55% city/45% highway) baseline average speed (29.1 mph, or 46.8 kph) is calculated as the weighted harmonic average of the city and highway driving speeds, shown in Supplementary Equation (4).

$$Combined\ Speed = \frac{1}{\left(\frac{0.55}{On-road\ City\ Driving\ Speed}\right)+\left(\frac{0.45}{On-road\ Highway\ Driving\ Speed}\right)} \quad (4)$$

Supplementary References

1 EPA (U.S. Environmental Protection Agency), 2006. Fuel Economy Labeling of Motor Vehicle Revisions to Improve Calculation of Fuel Economy Estimates, Final Technical Support Document. Report EPA420-R-06-017, Washington, DC. Retrieved from http://www3.epa.gov/carlabel/documents/420r06017.pdf (accessed June 6, 2018)

2 Elgowainy, A. et al. (2016). Cradle-to-Grave Lifecycle Analysis of US Light Duty Vehicle Fuel Pathways: A Greenhouse Gas Emissions and Economic Assessment of Current (2015) and Future (2025-2030) Technologies (No. ANL/ESD-16/7). Argonne National Lab (ANL), Argonne, IL (United States).

3 Argonne National Laboratory. GREET.Net Database (Argonne National Laboratory, Lemont, Illinois, USA, 2017).

4 U.S. Life Cycle Inventory Database. (2012). National Renewable Energy Laboratory, 2012. Retrieved from https://www.lcacommons.gov/nrel/search (accessed August 3, 2018)
